# Neonatal Postresuscitation Care in Brazil: A National Overview

**DOI:** 10.1055/a-2781-4614

**Published:** 2026-01-21

**Authors:** João Cesar Lyra, Ligia Maria S.S. Rugolo, Leni Marcia Anchieta, Ruth Guinsburg, Maria Fernanda Branco de Almeida

**Affiliations:** 1 Department of Pediatrics, UNESP, Univiversidade Estadual Paulista, Sao Paulo State, Brazil; 2 Department of Pediatrics, School of Medicine, Federal University of Minas Gerais, Belo Horizonte, Brazil; 3 Division of Neonatal Medicine, Escola Paulista de Medicina - Universidade Federal de São Paulo, Brazil

**Keywords:** newborn, patient care planning, resuscitation, survey

## Abstract

**Objectives:**

Postresuscitation care (PRC) encompasses structured and systematic interventions aimed at promptly stabilizing at-risk newborns in order to improve clinical outcomes. This study aimed to assess PRC practices as reported by pediatricians who serve as instructors in the Brazilian Neonatal Resuscitation Program (BNRP) of the Brazilian Pediatric Society.

**Methods:**

We conducted a cross-sectional, descriptive survey among BNRP instructors. Data were collected via a 55-item online questionnaire (Google Forms), covering respondents' professional background, primary work setting, and specific PRC practices. A convenience sample was used, and descriptive statistics summarized the findings.

**Results:**

A total of 740 responses were obtained, representing 63% of BNRP instructors. Of these, 79% were neonatologists, 88% with over 10 years of professional experience. Most worked in public (61%) and teaching hospitals (76%). Only 41% had received targeted PRC training; of these, 56% had exclusively theoretical instruction. Regarding the scope of PRC, 37% believed interventions were indicated solely for newborns requiring intubation, chest compression, or medications in the delivery room. Overall, 49% of respondents reported having written PRC protocols at their institutions, though their content and implementation varied considerably.

**Conclusion:**

PRC practices in Brazil are neither homogeneous nor systematically implemented across most neonatology services involving BNRP instructors. These findings highlight the pressing need for enhanced dissemination of standardized PRC protocols and comprehensive training for pediatricians engaged in neonatal care.

**Key Points:**

- PRC can improve outcomes in high-risk newborns, but its real-world application remains poorly described in middle-income countries.
- This is the largest study to date on PRC practices, based on responses from instructors of the Brazilian Neonatal Resuscitation Program.
- Findings reveal the need for structured training and underscore the importance of further research on the impact of standardized PRC on neonatal outcomes.

## Introduction

The “Golden Hour” in neonatal care refers to the comprehensive management of newborns during their first hour of life, encompassing procedures during and after delivery room resuscitation to achieve early stabilization of respiratory, cardiovascular, neurological, and metabolic functions. 1 2 3 Extending this concept, so-called postresuscitation care (PRC) has emerged as a critical component of neonatal care, which involves standardized, evidence-based interventions delivered by qualified professionals with appropriate resources. 4 5

Key PRC objectives include maintaining normothermia, optimizing oxygen saturation, implementing non-invasive respiratory support, applying neuroprotective measures, preventing hypoglycemia and infection, and ensuring stability upon neonatal intensive care unit (NICU) admission. Furthermore, PRC includes the active involvement of the family in the care process, always striving for effective communication among all those involved in the care of the newborn. 6

Implementing these concepts has shown benefits in improving prognoses for high-risk newborns, including preterm infants and those requiring resuscitation, irrespective of gestational age (GA). 7 Multiple studies confirm that early stabilization protocols for at-risk newborns improve patient outcomes. 8 9

Although many aspects of the neonatal “Golden Hour” align with International Liaison Committee on Resuscitation (ILCOR) recommendations, 10 11 global implementation in routine neonatal care is not well-documented, with reported inconsistencies across healthcare services. 12 13 This study aimed to investigate the implementation of postresuscitation stabilization practices for high-risk newborns in Brazilian neonatal units, from the perspective of Brazilian Neonatal Resuscitation Program (BNRP) instructors.

## Materials and Methods

We conducted a cross-sectional study from November 1 to December 31, 2023. Pediatricians certified to be BNRP instructors, from all five regions of the country, were invited to participate voluntarily and anonymously. In Brazil, only medical doctors are permitted to serve as BNRP instructors.

An online 55-question questionnaire, created using Google Forms, was distributed via email and WhatsApp by the BNRP coordination to all 1,174 certified instructors. Participants accessed the questionnaire after reading and accepting the Consent Form, which contained the completion instructions. Considering that the participants were pediatricians and neonatologists who served as instructors in the BNRP, it was assumed that, due to their extensive access to current recommendations, these professionals had comprehensive knowledge on the subject and were familiar with this type of instrument. The questionnaire underwent a pilot test conducted by the BNRP executive group, neonatologists, and pediatricians to ensure its ease of use.

The questionnaire collected demographic data, hospital characteristics, and routine practices during the first hours after birth. We assumed that participants were familiar with PRC concepts and recommendations, as these had been established and disseminated by the BNRP manual published in 2018. Questions were multiple-choice or open-ended, allowing for single or multiple responses. Key topics included characteristics of participants and their primary workplaces, features of hospital PRC, and specifics of care in the delivery room, during intra-hospital transport, and in the NICU. The questionnaire included a specific section to assess the infrastructure of participants' workplaces, aiming to evaluate the continuity of care after resuscitation procedures in the delivery room. Additionally, several questions addressed the care of very low birth weight preterm infants, given that approximately 70% of these newborns require resuscitation at birth in Brazil. 14 The questionnaire was developed in accordance with the Checklist for Reporting Results of Internet E-Surveys (CHERRIES) guidelines 15 and is available as a Supplement (available in the online version only). Data collection concluded after 60 days, yielding responses from all Brazilian regions and states, thus forming a convenience sample.

The study received approval from the Ethics Committee of the leading institution (approval no.: 71541023.3.0000.5411). The Free and Informed Consent Term was presented on the form's first page.

### Statistical Analysis

Survey responses were expressed as a number or proportions. Data were summarized as descriptive statistics using Sigma Plot (Systat Software, San Jose, California, United States).

## Results

We obtained 740 responses, representing 63% of all certified BNRP instructors across Brazil's 27 federative units. All questionnaires were fully completed. Most respondents (79%) were neonatologists, 88% with over 10 years of clinical practice, and 91% work with critically ill newborns. Their primary workplaces were predominantly public (61%) and teaching hospitals (75%). Participant demographics and professional activities are detailed in Table 1 .

**Table 1:** Characteristics of instructors and their respective hospital settings

Survey question	Responses, *n* = 740 (%)
Regions of the country	
North	92 (12)
Northeast	190 (26)
Midwest	65 (9)
South	103 (14)
Southeast	290 (39)
Type of hospital	
Public	453 (61)
Private	112 (15)
Both	175 (24)
Teaching hospital	559 (76)
Gestational age usually attended in the delivery room	
≥34 weeks	29 (4)
<34 weeks	17 (2)
Any gestational age	694 (94)
Participants' area of expertise	
Neonatologist	584 (79)
Pediatrician	109 (15)
Pediatric intensive care physician	47 (6)
Years of experience	
>15	501 (68)
10–15	145 (20)
5–9	83 (11)
<5	11 (1.0)
Training in postresuscitation care	302 (41)

**Fig. 1:**
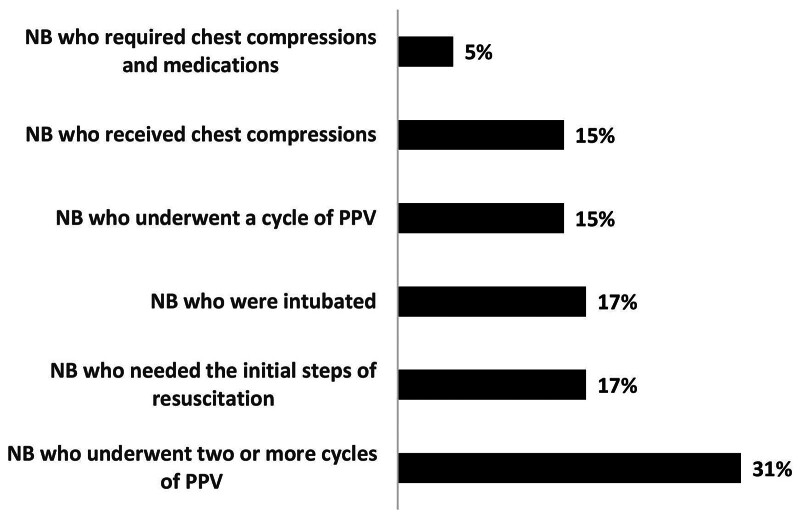
Indications of the postresuscitation care based on the opinion of the participants (%). NB, newborn; PPV, positive pressure ventilation.

Only 41% of participants reported receiving PRC training, with 56% indicating it was exclusively theoretical. Regarding PRC indications, 37% of instructors believed these measures were exclusively for newborns requiring intubation, chest compressions, or resuscitation medication in the delivery room ( Fig. 1 ).

A written PRC protocol was reported by 49% of respondents at their primary workplace. Table 2 details the aspects covered in these protocols and specific practices across different settings, including the delivery room (DR), intra-hospital neonatal transport, and the NICU. Responses concerning the typical locations for CRP within hospitals varied based on each facility's characteristics, including the delivery room, “recovery room,” “observation room,” “stabilization room,” and “neonatal unit.”

**Table 2:** Characteristics of postresuscitation care at the participants' primary workplaces

Survey question	Responses, *n* = 740 (%)
Specific written protocols on PRC in hospital settings	361 (49)
If there is a protocol, what is its scope? a	
Thermoregulation in the DR	349 (97)
Thermoregulation in the NICU/another setting	292 (81)
Respiratory stabilization in the delivery room	331 (92)
Intra-hospital neonatal transport	332 (92)
Respiratory stabilization in the NICU/another setting	289 (80)
Initial supply of fluids, electrolytes, and glucose	311 (86)
Nutritional approach on the first day of life	280 (76)
Addressing infectious risk	300 (83)
Usual setting for newborn stabilization	
NICU/neonatal mid-risk unit	559 (75)
Another hospital settings	181 (25)
Common practices on postresuscitation care in the DR	
Newborn's temperature monitoring	607 (82)
CPAP use	683 (92)
Elective intubation according to GA (wk)	216 (29)
<32	20 (3)
<30	19 (3)
<28	93 (13)
<26	84 (11)
Common practices during intra-hospital transportation	
Thermoregulation (more than one answer allowed)	
Neonatal transport incubator	686 (93)
Cap	694 (94)
Plastic wrap	705 (95)
Thermal mattress	142 (19)
Heated gases	133 (18)
Equipment commonly used for pulmonary ventilation	
T-piece resuscitator	486 (66)
Self-inflating bag	146 (20)
Electronic transport ventilator	106 (14)

Abbreviations: CPAP, continuous positive airway pressure; DR, delivery room; GA, gestational age; NB, newborn; NICU, neonatal intensive care unit; PRC, postresuscitation care.

Note: ^a^ *n* = 361.

### Care Practices Provided in the Delivery Room

**Fig. 2:**
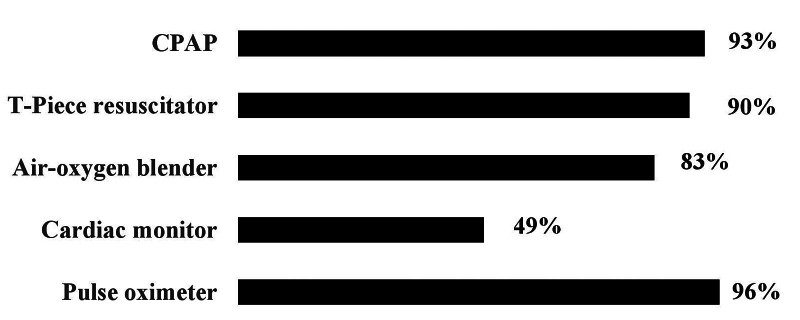
Availability of equipment for monitoring and respiratory assistance in the delivery room (%). CPAP, continuous positive airway pressure.

Temperature monitoring in the DR was reported by 82% of participants (607/740); 87% (527/607) applied this to all newborns, while 13% (80/607) restricted it to preterm infants (<37 weeks' GA). Early continuous positive airway pressure (CPAP) was considered by 92% (683/740), with 78% applying it to any newborn with respiratory distress. Prophylactic CPAP for newborns <32 weeks' GA was recommended by 4% (33/740), and by 9% (70/740) for those <34 weeks' gestation. Elective intubation based solely on gestational age was indicated by 29% of participants. Most workplaces had recommended monitoring and respiratory assistance equipment in the DR, but cardiac monitors were an exception, present in only 49% of cases ( Fig. 2 ).

### Standards of Care in the Context of Intrahospital Transportation

All participants reported measures to maintain newborn temperature during intra-hospital transport. Over 90% cited basic thermoregulation requirements. The majority use a resuscitator with a T-piece for respiratory support, but only 18% reported using heated gases ( Table 2 ).

### Initial Care Practices Provided to Preterm Newborns <34 Weeks' Gestation in the NICU

Only 55% of respondents reported a readily available and equipped hospital bed for newborn admission. For vascular access, 47% lacked a specific protocol for venous access indication, with most recommending umbilical artery catheterization for clinically unstable neonates. The first surfactant dose is administered in the NICU in 86% of cases, and in 7% in the DR. Caffeine is prescribed within 24 hours of life by 88% of respondents (650/740), with 90% (587/650) administering it prophylactically, mainly for newborns <32 weeks' gestation. Table 3 provides detailed results.

**Table 3:** Initial care practices for newborns less than 34 weeks' gestation

Survey question	Responses, *n* = 740 (%)
Aspects of care on admission to the neonatal unit	
Bed designated for patient admission is always ready and suitable	405 (55)
Temperature monitoring of the newborn is consistently assessed	727 (98)
First venous vascular access preferably obtained	
Umbilical vein	332 (45)
Peripheral vein	32 (4)
Peripherally inserted central catheter (PICC)	31(4)
Not defined in advance	345 (47)
Indications for umbilical artery catheterization	
In all cases	20 (3)
Never performed	172 (23)
In clinically unstable NB	548 (74)
Time of administration of the first dose of surfactant	
In the first 2 hours of life In the first 6 hours of life Over 6 hours old No pre-set time	544 (74) 144 (19) 44 (6) 8 (1)
Administration technique of surfactant	
Tracheal tube INSURE Thin catheter (LISA/MIST)	276 (37) 297 (40) 167 (23)
Use of caffeine in the first 24 hours of life	650 (88)
Blood pressure monitoring	537 (73)
There is a specific protocol for dealing with hypotension	296 (40)
Use of antibiotics in cases of suspected EOS	
According to the NB's clinical condition, in association with risk factors for infection and laboratory test results	368 (50)
Nutritional approach (more than one answer allowed)	
Oropharyngeal colostrum therapy	527 (71)
Early enteral nutrition	599 (81)
Parenteral nutrition	466 (63)
Intravenous amino acid solution use	322 (44)

Abbreviations: EOS, early-onset sepsis; INSURE, “intubate-surfactant administration-extubate to CPAP”; LISA, less invasive surfactant; MIST, minimally invasive surfactant; NB, new born.

Regarding the hemodynamic management of newborns, 73% reported routinely measuring blood pressure in all patients upon admission to the unit, but only 40% had a protocol for hemodynamic disturbances. Echocardiographic assessment was mentioned by 21%. For suspected early-onset neonatal sepsis, 50% prescribe antibiotics based on clinical status, in conjunction with risk factors and lab findings, while 43% use clinical condition and risk factors alone; 7% do not consider clinical presentation. Notably, 40% (300/740) reported no specific protocols for early neonatal infection management at their workplaces.

Most participants recommended early enteral nutrition. However, 9% reported keeping even clinically stable extremely preterm newborns fasting for the first 24 hours.

### Teamwork and Communication with the Family

Interprofessional communication within the healthcare team was rated good but with improvement aspects by 52% of instructors, appropriate by 41%, and not appropriate by 7%. Family participation in care occurred “wherever possible” for 77%, “only when expressed desire” for 13%, and “never involved” for 10%.

A positive response regarding the presence and use of an essential care protocol (minimum handling) for preterm newborns was given by 79%; however, 11% reported the protocol was in place but not adhered to, and 10% reported no such protocol.

## Discussion

Postresuscitation care is crucial in neonatal practice, significantly reducing morbidity and mortality in at-risk newborns. 3 4 5 The BNRP of the Brazilian Society of Pediatrics recommendations, aligned with international consensus, emphasize the importance of its implementation; however, the application of this strategy in Brazilian neonatology services lacks systematic evaluation. This pioneering study addresses this gap by investigating the practice through a questionnaire directed at certified BNRP instructors.

We selected BNRP instructors due to their presumed deep knowledge of clinical workflows and leadership roles. It is noteworthy that in Brazil, BNRP instructors are exclusively physicians, predominantly neonatologists, unlike some other countries where non-physicians may also serve. 14 The robust 63% response rate from public and private hospitals across all five regions ensures a representative national overview of the national landscape.

Our findings revealed limited clarity among instructors regarding PRC indications and considerable variability in practice, likely reflecting the absence of specific guidelines. Although ILCOR recognizes the importance of CPR, there were no specific recommendations at the time this research was conducted, which may have contributed to the heterogeneity observed. Another important consideration is that fewer than half of instructors reported prior PRC training, mostly theoretical, highlighting the need for targeted education. Although we did not assess training impact, previous research shows that neonatal care training improves skill acquisition and protocol adherence. 16 17 18 19 20

Alarmingly, half of the participants reported no written PRC protocol at their primary workplace. Even among those with protocols, some lacked explicit guidance on respiratory stabilization or nutritional management. This is critical, as structured, evidence-based protocols demonstrably improve care quality, reduce medical errors, prevent complications, and lower hospital costs. 21 Their positive impact extends to neonatal outcomes, reducing hypothermia, hypoglycemia, improving vascular access, fluid administration, and even decreasing bronchopulmonary dysplasia (BPD) risk. 3 9 22 23 A recent meta-analysis assessing the effects of Golden Hour care on early clinical outcomes and prematurity-related comorbidities demonstrated reductions in hypothermia rates at NICU admission and at 1 hour of life, shorter times to initiation of fluid therapy and administration of the first surfactant dose, and a decreased incidence of severe intraventricular hemorrhage. 24

Considering that the early stabilization of the neonate following resuscitation should occur in a systematic and organized manner, it is reasonable to speculate that the procedures encompassed by PRC are more likely to be performed in settings equipped with appropriate technology, resources, and trained personnel, such as intermediate care or neonatal intensive care units. Although no studies have evaluated neonatal outcomes comparing different locations where PRC is provided, it is plausible that such factors may influence the prognosis of at-risk newborns. In our study the initial management of at-risk neonates does not necessarily occur in intermediate or high-risk care units, highlighting the heterogeneity of neonatal care practices. Nevertheless, this is a concerning finding, as there is no assurance that these locations have the necessary infrastructure to provide safe and adequate care. 25

Regarding specific clinical practices, although nearly all participants reported having PRC protocols, including thermoregulation measures, almost 20% did not monitor temperature in the delivery room, highlighting the gap between theoretical guidelines and actual practice. This discrepancy aligns with data from the Brazilian Neonatal Research Network, which reported high admission hypothermia rates (20–93%) in Brazilian university-affiliated tertiary public hospitals. 26 27

Our study identified variability in respiratory support, with limited CPAP use for stabilizing preterm infants <34 weeks and intubation decisions based solely on gestational age. This contradicts strong evidence supporting early CPAP to avoid intubation, which positively impacts preterm outcomes by reducing mechanical ventilation, BPD, and the composite outcome of BPD or death. 28 29

For preterm infants <34 weeks' gestation, the majority of survey respondents administer prophylactic caffeine within the first 24 hours and provide surfactant within 2 hours, in line with national and international guidelines. 30 31 32 33 34 However, the adoption of the thin catheter technique (LISA/MIST) remains limited, as also reported in an Australian survey. 13

Regarding hemodynamic control, arterial blood pressure monitoring in premature neonates is a common practice. However, the majority of participants reported not having a protocol for managing hypotension, which raises concerns about both variability and the potential for overtreatment or undertreatment with vasoactive drugs. 35 With respect to antibiotic use, most participants reported the absence of a protocol for managing newborns at risk of early infection, which is a concern since guidelines for early-onset sepsis help identify those requiring treatment and prevent unnecessary antibiotic exposure. 20 36 37 38

Nutritional management of preterm infants also requires improvement. Although early enteral nutrition is widely recommended, some participants still advise fasting for clinically stable extremely preterm newborns in the first 24 hours and delaying the initiation of amino acid solutions. This contrasts with robust evidence demonstrating the benefits of standardized nutritional protocols on growth and reducing risks like necrotizing enterocolitis, late-onset sepsis, BPD, and neurological impairment. 39 40

Finally, the core concepts of PRC encompass other relevant aspects with prognostic impact, such as the organization of care, communication, and integration among healthcare team members and with family. In this study, just over half of the participants reported a fully prepared bed for newborn admission, suggesting organizational gaps and/or inefficient communication among team members. Concerning family integration, the majority reported that family involvement in neonatal care is not routine and occurs only when feasible, indicating an opportunity for improvement in this aspect. 2 23

## Limitations and Strengths

This study is based on self-reported practices, which may not always reflect actual clinical performance. Nonetheless, the high participation of experienced neonatologists from academic centers enhances the credibility of the data. Although it did not evaluate the direct impact on neonatal outcomes, this is the first study on PRC conducted in Brazil and, to our knowledge, the largest PRC-focused study published to date. A limitation is that only neonatal resuscitation BNRP instructors were recruited, which may not fully represent routine practice nationwide, especially given the country's large geographic size and the considerable heterogeneity in neonatal care delivery across regions. Moreover, this study focused on the general aspects of PRC and did not explore specific clinical conditions such as hypoxic–ischemic encephalopathy, meconium aspiration, or pneumothorax. Likewise, we did not evaluate details related to the type of monitoring employed, the collection of routine laboratory tests, or the provision of counseling regarding prognosis, which could represent relevant elements in the comprehensive management of these patients.

There may also have been a bias toward tertiary and secondary care units, as most participants were neonatologists rather than general pediatricians. However, the inclusion of highly trained professionals with extensive experience in neonatal care adds strength to the validity of the findings. If these practices reflect conditions in better-resourced teaching hospitals, the situation is likely even more limited in less specialized settings.

## Conclusion

Our study demonstrates that although neonatal PRC practices are broadly adopted by most BNRP instructors, significant heterogeneity exists in unit infrastructure, care practices, and protocol development and adherence. Our results highlight critical opportunities to enhance the quality of early newborn stabilization in Brazil, which could be partially addressed through targeted training focused on PRC.
