# Supplementary material for: Neonatal Postresuscitation Care in Brazil: A National Overview
Source: Am J Perinatol. 2026 Jan 21;43(11):1465–71. doi: 10.1055/a-2781-4614 (PMC13406385; doi:10.1055/a-2781-4614)
Supplement: Supplementary file 1 — Supplementary Material [file 10-1055-a-2781-4614_29248417.pdf]

SUPLLEMENT

Checklist for Reporting Results of Internet E-Surveys (CHERRIES)

Checklist Item	***Explanation***	***Page Number***
**Describe survey design**	Convenience sampling.	**1-2** (main text)
**IRB approval**	Ethics Committee of the leading Institution (Nº: 71541023.3.0000.5411)	**1-2** (main text)
**Informed consent**	A Free and Informed Consent Term was available for reading and acceptance on the first page of the form.	**4-5** (Suppl.)
**Data protection**	No personally identifiable information was collected. Confidentiality and privacy aspects are described in the Free and Informed Consent Term.	**4-5** (Suppl.)
**Development and testing**	To ensure ease of use and technical functionality, the electronic questionnaire underwent a pilot test by members of the BRAZILIAN NEOANATAL RESUSCITATION PROGRAM executive group, along with a selection of neonatologists and pediatricians.	**1-2** (main text)
**Open survey versus closed survey**	This was a closed survey; the link was made available exclusively to all certified instructors by the BRAZILIAN NEOANATAL RESUSCITATION PROGRAM coordination.	**1-2** (main text)
**Contact mode**	The link to access the form was made available by the BRAZILIAN NEOANATAL RESUSCITATION PROGRAM coordination to all certified instructors of the program via e-mail and WhatsApp.	**1-2** (main text)
**Advertising the survey**	All BRAZILIAN NEOANATAL RESUSCITATION PROGRAM instructors received an invitation to take part in the survey via their email or WhatsApp contact.	**1-2** (main text)
**Web/E-mail**	Google Forms was used to capture responses. Google Forms streamlines the process of data collection by instantly saving responses, organizing them neatly into a spreadsheet for detailed analysis, and providing immediate visual summaries.	**1-2** (main text)
**Context**	The survey was not posted on any specific website. The Google Forms link was sent to each respondent.	**1-2** (main text)
**Mandatory/voluntary**	Participation was voluntary.	**1-2** (main text)
**Incentives**	No incentives of any kind were offered to the research participants. This was described in the Free and Informed Consent Term.	**4-5** (Suppl.)
**Time/Date**	Data were collected from November 1st to December 31st, 2023.	**1-2** (main text)
**Randomization of items or questionnaires**	Not applicable. There was no randomization of questionnaire items.	**-**
**Adaptive questioning**	Most of the questions required direct answers; few items were conditioned on previous answers.	**6-12** (Suppl.)
**Number of Items**	The questionnaire contained 55 questions, with an average response time of 15 minutes.	**6-12** (Suppl.)

**Number of screens (pages)**	The form comprises six sections, corresponding to 18 pages in PDF format.	**6-12** (Suppl.)
**Completeness check**	All questions in the form were mandatory, preventing participants from proceeding without answering every item.	**6-12** (Suppl.)
**Review step**	Respondents were able to review and change answers before submission.	**-**
**Unique site visitor**	Not applicable. The survey was distributed via a direct link using Google Forms, which does not track IP addresses, cookies, or login credentials. As such, it was not possible to determine the number of unique site visitors in a technical sense. Participation was based solely on link recipients.	**-**
**View rate (Ratio of unique survey visitors/unique site visitors)**	Not applicable. The survey link was distributed directly via email and messaging apps to a predefined group of 1174 individuals, rather than posted on a public website. Therefore, site visitor data is not available.	**-**
**Participation rate (Ratio of unique visitors who agreed to participate/unique first survey page visitors)**	Not precisely measurable. Google Forms does not provide data on users who visited the first page but did not proceed. However, out of 1174 individuals who received the survey link, 740 completed the questionnaire (estimated participation rate ≈ 63%).	**-**
**Completion rate (Ratio of users who finished the survey/users who agreed to participate)**	The completion rate was 100%, as all participants who agreed to take part completed the questionnaire, answering all items. The questionnaire's formatting did not allow submission unless fully completed.	**6-12** (Suppl.) **1-2** (main text)
**Cookies used**	Cookies were not used to assign a unique user identifier to each computer.	**-**
**IP check**	Not applicable, as the IP addresses of participants' computers were not monitored.	**-**
**Log file analysis**	Responses were checked manually using participants' email addresses and demographic data, allowing the identification and exclusion of duplicate responses. Google Forms does not provide access to log files such as IP addresses or timestamps with sufficient detail for automated duplicate detection.	**-**
**Registration**	This is a closed survey. The link to access the form was made available exclusively to all certified instructors by the BRAZILIAN NEOANATAL RESUSCITATION PROGRAM coordination. Participants who agreed to take part registered their email addresses (a mandatory field). This enabled survey coordinators to check for duplicate responses, which did not occur.	**-**
**Handling of incomplete questionnaires**	The questionnaire was designed so that it could not be submitted without answering all questions. Thus, incomplete questionnaires were not possible.	**6-12** (Suppl.)
**Questionnaires submitted with an atypical timestamp**	Not applicable. This aspect was not evaluated in this study.	**-**

**Statistical correction**	No statistical correction was performed. Survey responses are expressed as numbers or proportions and summarized as descriptive statistics.	**2** (main text)

This checklist has been modified from Eysenbach G. Improving the quality of Web surveys: the Checklist for Reporting Results of Internet E-Surveys (CHERRIES). J Med Internet Res. 2004 Sep 29;6(3):e34 [erratum in J Med Internet Res. 2012; 14(1): e8.]. Article available at https://www.jmir.org/2004/3/e34/; erratum available https://www.jmir.org/2012/1/e8/. Copyright ©Gunther Eysenbach. Originally published in the Journal of Medical Internet Research, 29.9.2004 and 04.01.2012.

This is an open-access article distributed under the terms of the Creative Commons Attribution License (https://creativecommons.org/licenses/by/2.0/), which permits unrestricted use, distribution, and reproduction in any medium, provided the original work, first published in the Journal of Medical Internet Research, is properly cited.

INFORMED CONSENT FORM

Research Project Title: Neonatal Post-Resuscitation Care in Brazil: A National Overview.

This document serves to invite you to participate in a study on post-resuscitation care practices in newborns (NB) in Brazil.

Post-resuscitation care in the neonatal period can be defined as a systematic and organized approach to evaluating and supporting the respiratory, cardiovascular, neurological, renal, gastrointestinal, metabolic, and hematological systems, according to each patient's needs. Although monitoring and clinical stability of the newborn in the first hours of life are essential factors for a good prognosis, the application of post-resuscitation care in Brazil is not fully understood. In this context, the objective of this research is to investigate the practices carried out in Neonatology services across the country.

To participate in the study, you will receive a link to complete an online questionnaire, formatted via "Google Forms," with objective questions about the care practices at your primary workplace.

The questionnaire consists of 55 questions, with an average completion time of 15 minutes.

Your participation in this research can provide valuable information for us to better understand the Brazilian reality concerning this issue. The data obtained will be useful for future planning and the development of strategies for knowledge dissemination and training in post-resuscitation care.

The information obtained will be analyzed collectively with that of other participants. Under no circumstances will your name or identification be disclosed. Confidentiality and your privacy are guaranteed by the researchers, who commit to using the collected data solely for research purposes. It is important for you to know that there will be no personal expenses for the study participant, and no payment will be made to you for your participation. Participants have the right to seek compensation for any damage resulting from the research. This research presents minimal risks to the participant, which will be mitigated through careful handling and confidentiality of information based on the General Data Protection Law (LGPD). No specific intervention will be performed.

You have the right to be kept updated on the study results. Your authorization is voluntary, and you may freely withdraw it at any time, should you so desire, without any prejudice to you.

At any stage of the study, you will have access to the professionals responsible for the research to clarify any doubts. You can contact the researchers listed below or the Research Ethics Committee of FMB-UNESP, by calling (14) 3880-1608 or 3880-1609. The committee operates Monday through Friday, from 8:00 AM to 11:30 AM and from 2:00 PM to 5:00 PM, at Chácara Butignolli s/nº in Rubião Júnior – Botucatu – São Paulo.

After approval by the Research Ethics Committee (CEP), this document will be attached to the online form. Upon reading and understanding it, and having accepted its terms, you may begin answering the questions. This document will be kept on file by the researchers for five years, and if you wish, you may request a copy signed by the researcher for your own records.

If you believe you have been sufficiently informed about the content of this document, by accepting to participate in the research, you will be directed to the questionnaire.

Researcher Contact Information:

Ligia Maria S.S. Rugolo – e-mail: ligia.rugolo@unesp.br

João Cesar Lyra – e-mail: joao.lyra@unesp.br

Depto. de Pediatria da Faculdade de Medicina de Botucatu UNESP– Campus Rubião Júnior.

Department of Pediatrics, Botucatu Medical School, São Paulo State University (UNESP) – Rubião Junior Campus

Fone: 14- 3880 1483 / 14-3811 6274

Questionnaire

Title: Neonatal Post-Resuscitation Care in Brazil: A National Overview

Total number of questions: 55

Estimated response time: 15 minutes

Section I: Participant and Workplace Characteristics

1. **Gender:**
   - Male
   - Female
   - Prefer not to disclose
2. **Age:**
3. **City of residence:**
4. **State of residence:**
5. **Years since medical graduation:**
6. **Medical specialty / Area of practice:**
   - Pediatrician
   - Neonatologist
   - Pediatric intensivist
7. **In which city is your primary workplace located?**
8. **In which state is your primary workplace located?**
9. **Type of hospital where you primarily work:**
   - Public
   - Private
   - Both
10. **Is your main hospital a teaching hospital?**
    - Yes
    - No
11. **Areas of neonatal care in which you work (select all that apply):**
    - Delivery room
    - Rooming-in
    - Neonatal Intensive Care Unit (NICU)
    - Intermediate Care Unit
    - Kangaroo Unit
    - Combined Pediatric-Neonatal ICU
12. **How frequently do you work in the delivery room?**
    - Daily
    - Once a week

- More than once a week
- Once a month
- Occasionally
- I do not work in the delivery room

13. **If you work in the delivery room, what is the usual gestational age of the newborns you attend?**
    - Only ≥34 weeks
    - Only <34 weeks
    - Any gestational age

14. **Which neonatal resuscitation courses are you an instructor for? (Select all that apply):**
    - Neonatal Resuscitation Program
    - Preterm Newborn Resuscitation in the Delivery Room
    - High-Risk Newborn Transport Course

Section II: Post-Resuscitation Care Practices in Your Main Workplace

15. **For which newborns is post-resuscitation care indicated in your service? (Select all that apply):**
    - Newborns who underwent two or more cycles of PPV
    - Newborns who required only initial resuscitation steps
    - Newborns who were intubated
    - Newborns who received a single cycle of PPV
    - Newborns who received chest compressions
    - Newborns who required chest compressions and medications

16. **Is there an established protocol for neonatal post-resuscitation care in your service?**
    - Yes
    - No

17. **If you answered YES, which of the following components are included in your protocol? (Select all that apply):**
    - Maintenance of normothermia in the delivery room
    - Respiratory stabilization in the delivery room
    - Transport from the delivery room to the neonatal unit
    - Maintenance of normothermia upon NICU admission
    - Respiratory stabilization upon NICU admission
    - Initial fluid, electrolyte, glucose, and hemodynamic stabilization
    - Nutritional approach during the first day of life
    - Infection risk assessment and management
    - Others (specify): ______________________________

18. **Where is post-resuscitation care usually provided in your service?**
    - NICU

- Delivery room
- Other (specify): ______________________________

19. **Have you received any training on neonatal post-resuscitation care?**
    - Yes
    - No
20. **If YES, what type of training did you receive?**
    - Theoretical only
    - Theoretical and practical
    - Practical only
21. **Would you like to receive training on neonatal post-resuscitation care?**
    - Yes
    - No

Section III: Specific Practices in the Delivery Room – Routine Neonatal Care

22. **Do you use a blender to provide intermediate oxygen concentrations to newborns in the delivery room?**
    - Yes
    - No
23. **Do you use pulse oximetry for monitoring newborns in the delivery room?**
    - Yes
    - No
24. **Regarding supplemental oxygen delivery in the delivery room, do you adjust it according to target oxygen saturation levels?**
    - Yes
    - No
25. **Do you use a cardiac monitor for newborn monitoring in the delivery room?**
    - Yes
    - No
26. **Is a T-piece resuscitator available in your delivery room?**
    - Yes
    - No
27. **If a T-piece resuscitator is available, in what situations is it used?**
    - Only for newborns <34 weeks of gestation
    - For all newborns requiring positive pressure ventilation
28. **Is the newborn temperature monitored in the delivery room?**
    - Yes
    - No

29. **Which routine measures are used to maintain normothermia in preterm newborns in the delivery room? (Select all that apply):**
    - Room temperature ≥ 23°C
    - Care provided under a radiant heat source
    - Use of double cap
    - Plastic wrap
    - Thermal mattress
    - Heated gases

30. **What is your service's routine regarding CPAP use in the delivery room?**
    - CPAP for all newborns <34 weeks
    - CPAP for all newborns <32 weeks
    - CPAP for newborns with signs of respiratory distress
    - CPAP is not used in the delivery room

31. **If CPAP is used in the delivery room, what is the preferred type?**
    - Bubble CPAP
    - T-piece CPAP
    - Ventilator-generated CPAP

32. **If CPAP is used in the delivery room, what interface is most frequently used?**
    - Double nasal prong
    - Nasal mask
    - Face mask
    - Other (specify): ______________________________

33. **What is your service's routine regarding tracheal intubation in the delivery room?**
    - Elective intubation for all newborns with any degree of respiratory distress
    - Intubation only in case of failure or ineffectiveness of mask PPV
    - Elective intubation depending on gestational age
    - Elective intubation for surfactant administration

34. **If elective tracheal intubation is performed based on gestational age, what is the gestational age threshold?**
    - Newborns <32 weeks
    - Newborns <30 weeks
    - Newborns <28 weeks
    - Newborns <26 weeks
    - Elective intubation is not performed

Section IV: Specific Practices During Transport to the NICU

35. **Which routine measures are used to maintain normothermia in preterm newborns during transport to the NICU? (Select all that apply):**
    - Use of double cap
    - Plastic wrap
    - Thermal mattress

• Heated gases
• Transport incubator with double wall

36. **If the newborn was intubated in the delivery room, how is ventilation performed during transport?**
• Manual ventilation using a T-piece resuscitator
• Manual ventilation using a self-inflating bag
• Ventilation using a transport ventilator
• Ventilation by other means (specify): ______________________________

Section V: Specific Practices in the NICU – Routine Neonatal Care

37. **Upon admission to the NICU, how would you rate the readiness of the preterm newborn's bed (considering temperature adjustments, monitoring equipment, devices for initial care, respiratory support, etc.)?**
• The admission bed is always ready and properly equipped
• The admission bed is never ready or properly equipped

38. **Who receives the newborn in the NICU?**
• Physician and nurse
• Physician and nursing technician
• Physician, nurse, and nursing technician
• Physician, nurse, and physical therapist
• Physician, nurse, nursing technician, and physical therapist
• Nurse and nursing technician
• Nurse, nursing technician, and physical therapist

39. **Is the newborn's admission temperature routinely measured in the NICU?**
• Yes
• No

40. **Where is surfactant therapy usually administered in your service?**
• Always in the delivery room
• Preferably in the delivery room
• Occasionally in the delivery room
• Never in the delivery room
• Preferably in the NICU
• Always in the NICU

41. **Regarding the timing of surfactant therapy, when is it most often administered?**
• Within the first hour of life
• Within the first two hours of life
• Within the first six hours of life
• Anytime within the first 24 hours of life

42. **What is the preferred method of surfactant administration in your service?**
• Thin catheter technique (MIST/LISA)

• INSURE
• Via endotracheal tube, with invasive mechanical ventilation

43. **Is blood pressure routinely monitored upon NICU admission?**
• Yes
• No

44. **Is there a specific protocol for managing hypotension?**
• Yes
• No

45. **How are vasoactive drugs typically indicated in the NICU during the first six hours of life?**
• Based solely on clinical assessment of the newborn's hemodynamic status
• Based solely on echocardiographic findings
• Based on clinical assessment combined with echocardiographic findings
• There is no specific protocol for this indication
• Based on the attending physician's preference

46. **How are antibiotics typically prescribed in the NICU during the first six hours of life?**
• Based on clinical condition, risk factors for infection, and laboratory findings
• Based on clinical condition and risk factors
• Based only on risk factors and laboratory findings

47. **Regarding caffeine use on the first day of life, what is your service's routine? (Select all that apply):**
• Prophylactic use for all newborns <32 weeks' gestation
• Prophylactic use for all newborns <28 weeks' gestation
• Prophylactic use only for intubated newborns expected to be extubated within 24 hours
• Only for the treatment of apnea
• Caffeine is not used within the first 24 hours of life

48. **Regarding nutrition, what are the routine practices in your service during the first 24 hours of life? (Select all that apply):**
• Colostrum therapy
• Early enteral nutrition if clinically stable
• Prescription of intravenous amino acids
• Prescription of parenteral nutrition
• Fasting during the first 24 hours for extremely preterm infants
• Fasting only if enteral feeding is contraindicated due to clinical conditions

49. **Which of the following are routinely prescribed upon NICU admission for preterm newborns (intravenous infusion)? (Select all that apply):**
• Fluids and glucose
• Fluids, glucose, and calcium
• Fluids, glucose, calcium, and magnesium

- Fluids, glucose, electrolytes, and amino acids
- Parenteral nutrition

50. **What is the preferred initial vascular access for preterm newborns in your service?**
    - Umbilical vein
    - Peripheral vein
    - Peripherally inserted central catheter (PICC)
    - Depends on birth weight and clinical stability
    - Depends on attending physician's preference

51. **Where is the vascular access usually obtained?**
    - Always in the delivery room
    - Preferably in the delivery room
    - Occasionally in the delivery room
    - Never in the delivery room
    - Preferably in the NICU
    - Always in the NICU

52. **Under what circumstances is umbilical artery catheterization performed?**
    - Always
    - Never
    - Only for clinically unstable newborns
    - According to birth weight and gestational age

53. **How would you assess the quality of communication between the delivery room team and the NICU team?**
    - Adequate
    - Inadequate
    - Generally good, but with room for improvement

54. **Is there a protocol in your service for essential care (minimal handling) of preterm newborns?**
    - Yes
    - No
    - Yes, but it is generally not followed

55. **Regarding family involvement in the newborn's care during the first day of life, how would you describe your service's routine?**
    - As much as possible
    - Never
    - Only when the family expresses a desire to participate
